# Commensal Microbiome Expands Tγδ17 Cells in the Lung and Promotes Particulate Matter-Induced Acute Neutrophilia

**DOI:** 10.3389/fimmu.2021.645741

**Published:** 2021-03-29

**Authors:** Chorong Yang, Dong-il Kwon, Mingyu Kim, Sin-Hyeog Im, You Jeong Lee

**Affiliations:** ^1^ Department of Life Sciences, Pohang University of Science and Technology (POSTECH), Pohang, South Korea; ^2^ ImmunoBiome Inc., Pohang-si, South Korea; ^3^ Research Institute of Pharmaceutical Sciences, Seoul National University, Seoul, South Korea

**Keywords:** particulate matter, γδ T cell, neutrophilia, IL-17, commensal microbiome

## Abstract

Particulate matter (PM) induces neutrophilic inflammation and deteriorates the prognosis of diseases such as cardiovascular diseases, cancers, and infections, including COVID-19. Here, we addressed the role of γδ T cells and intestinal microbiome in PM-induced acute neutrophilia. γδ T cells are a heterogeneous population composed of Tγδ1, Tγδ2, Tγδ17, and naïve γδ T cells (TγδN) and commensal bacteria promote local expansion of Tγδ17 cells, particularly in the lung and gut without affecting their Vγ repertoire. Tγδ17 cells are more tissue resident than Tγδ1 cells, while TγδN cells are circulating cells. IL-1R expression in Tγδ17 cells is highest in the lung and they outnumber all the other type 17 cells such as Th17, ILC3, NKT17, and MAIT17 cells. Upon PM exposure, IL-1β-secreting neutrophils and IL-17-producing Tγδ17 cells attract each other around the airways. Accordingly, PM-induced neutrophilia was significantly relieved in γδ T- or IL-17-deficient and germ-free mice. Collectively, these findings show that the commensal microbiome promotes PM-induced neutrophilia in the lung *via* Tγδ17 cells.

## Introduction

γδ T cells are abundant in mucosal tissues, such as the conjunctiva, skin, and lung (1–3). Amongst them, Tγδ17 cells play an important role in the rapid response to foreign antigens by immediately secreting IL-17 and recruiting neutrophils into inflamed mucosal tissues (4, 5). In particular, the lungs are constantly exposed to various environmental insults such as airborne pathogens and inorganic pollutants. In this process, the commensal microbiome acts as an important immune modulator (6, 7). Recent studies have shown that dysregulated microbiota causes immune dysfunction, leading to poor control of respiratory infections, allergic asthma, or tumor immune surveillance (8–10). In the liver, the critical role of intestinal bacteria in the homeostasis of hepatic Tγδ17 cells has been described (11). However, the immune crosstalk between the commensal microbiota and lung-resident Tγδ17 cells has not yet been elucidated.

Air pollution is a serious problem worldwide, and accumulating evidence indicates that particulate matter (PM) has a significant effect on immune systems (12). Long-term exposure to PM induces IL-1 and TNFα secretion from alveolar macrophages (AM) and airway epithelial cells (AECs) (13–15) and is closely associated with increased mortality, morbidity, and hospitalization of asthma patients (16, 17). In particular, traffic-driven particles (diesel exhaust particles, DEPs) exacerbate house dust mite (HDM)-induced allergic asthma by enhancing Th17 cells in lungs (18, 19). In addition, recent studies have shown that the severity of air pollution is highly correlated with the mortality rate of COVID-19 patients; for every 1 μg/m3 increase in airborne fine dust concentration, the mortality of patients increases by 11% (20). To date, however, the role of γδ T cells in PM-induced pulmonary inflammation has not been well addressed.

γδ T cells are innate T cells that develop in the thymus. In a previous study, we showed that there are three distinct effector subsets in the thymus according to their transcriptional profiles designated as Tγδ1, Tγδ2, and Tγδ17 cells (21). These effector cells develop from common progenitors, and we showed that lineage differentiation models rather than TCR-mediated instructions and explained their ontogeny. In this study, we extended our previous research by analyzing γδ T cells in the peripheral lymphoid and non-lymphoid organs, including the lungs and defined their critical role in PM-mediated acute pulmonary neutrophilia. We defined naïve γδ T (TγδN) cells corresponding to conventional γδ T cells (CD44^lo^CD45RB^hi^) described previously (21, 22) and categorized all γδ T cells as TγδN, Tγδ1, Tγδ2, and Tγδ17 cells. Using anti-Vγ1, Vγ1/2, Vγ4, Vγ5, Vγ6, and Vγ7 antibodies in a single staining panel, we comprehensively analyzed Vγ TCR usage in all four sub-types of γδ T cells in peripheral tissues and compared them in specific pathogen-free (SPF) and germ-free (GF) mice. As a result, we found that commensal microbiota is critical for the maintenance of the peripheral pool of lung-resident IL-1R^+^ Tγδ17 cells, which contributed to the development of PM-induced acute airway neutrophilic inflammation, but not a chronic model of IL-17-dominant HDM/PM-induced allergic asthma. Consistent with this, PM-induced neutrophila was significantly relieved in GF mice compared to SPF mice. Collectively, these findings provide mechanistic insight into the immune crosstalk between commensal microbiome, lung-resident γδ T cells, and PM-induced neutrophilia.

## Methods

### Mice

B6 WT (C57BL/6) and *Tcrd ^-/-^* (B6.129P2-*Tcrd* ^tm1Mom/J^) mice were purchased from the Jackson Laboratory and bred in our facility under specific pathogen-free (SPF) conditions. *Il17a/f ^-/-^* (B6) mice were received from Dr. Charles D. Surh (POSTECH, Korea). CD45.1/2 B6 mice were received from Dr. Sin-Hyeog Im (POSTECH, Korea). All mice were used at the age of 6-12 weeks unless indicated, and age- and sex-matched animals were used as controls. Germ-free (GF) mice were bred and used as previously described (24). All mouse experiments were performed using protocols approved by the Institutional Animal Care and Use Committees (IACUC) of the POSTECH.

### PM-Induced Pulmonary Inflammation and Treatments

Mice were intranasally administered with 250 μg of particulate matter (PM) in saline or saline alone as controls. PM was obtained from Sigma (PM10-like ERMCZ100-1VL and ERMCZ120-1VL) and used as a 1:1 mixture. All intranasal administration (20 μl/nostril) was performed under anesthesia (i.p.) with ketamine (Yuhan)/xylazine (Rompun, BAYER) solution, as described (21).

### Mouse Models of Chronic HDM/PM-Induced Allergic Asthma

We used a previously described house dust mite (HDM)-induced mouse asthma model with minor modification (25). HDM (*Dermatophagoides pteronyssinus*) extracts were purchased from Greer laboratories. Mice were intranasally administered with 20 μg of HDM daily for 4 days and challenged again with 20 μg of HDM for 4 days later. Fine dust was administered *via* the intranasal route with 250 μg of PM.

### Parabiosis

Five-week-old B6 CD45.1/2 and CD45.2/2 female mice were joined together by parabiosis for 2 or 7 weeks, as previously described (11). Weight-matched mice were anesthetized and shaved. An incision was made along the side of each mouse and the skin was connected using surgical clips.

### Flow Cytometry and Antibodies

Single-cell suspensions were isolated and stained with fluorescein-conjugated antibodies. For cytokine detection experiments, lymphocytes were stimulated with Cell Stimulation Cocktail and protein transport inhibitors (eBioscience) for 4 hours. Cells were washed twice in FACS buffer and stained with surface markers for 30 min at 4°C. For intracellular staining, single-cell suspensions were surface-stained, fixed, and permeabilized with the eBioscience Foxp3 staining buffer set. Following antibodies were used; anti-CD4-BUV395 (BD, GK1.5), anti-CD8α-BV650 (BD, 53-6.7), anti-SiglecF-PE (BD, E50-2440), anti-CD11b-PerCP-Cy5.5 (BD, M1/70), anti-CD11c-PE-Cyanine7 (eBioscience, N418), anti-TCRβ-PE-CF594 (BD, H57-597), anti-Ly6G-APCCy7 (BD, IA8), anti-CD45.2-BV605 (BD, 104), anti-B220-BV711 (BD, RA3-6B2), anti-CD8-BV510 (BD, 53-6.7), anti-CD44-redFluor 710 (TONBO, IM7), anti-TCRβ-APCCy7 (BD, H57-597), anti-CD11c-BV650 (BD, HL3), anti-Thy1.2-BV786 (BD, 53-2.1), anti-GL3-BV421 (BD, GL3), anti-CD45.2-BV650 (Biolegend, 104), anti-CD11c-BV711 (BD, HL3), anti-GATA3-PE (ebioscience, TWAJ), anti-Tbet-PE-Cyanine7 (eBioscience, 4B10), anti-RORγ-PE-CF594 (BD, Q31-378), anti-PLZF- Alexa Fluor 647 (BD, R17-809), Zombie-Aqua (Biolegend), anti-RORγt- PerCP-Cy5.5 (BD, Q31-378), anti-IL-17A-BV650 (BD, TC11-18H10), anti-IFNγ-BV786 (BD, XMG1.2), anti-CD11b-BV711 (BD, M1/70), anti- IFNγ-PE (Invitrogen, XMG1.2), anti-proIL-1β- PE-Cyanine7 (Invitrogen, NJTEN3), anti-CD11c-APC (Invitrogen, N418), anti- CD121a (IL-1R, Type I/p80)-PE (Biolegend, JAMA-147), anti-IFNγ-BV421 (BD, XMG1.2), anti-GL3- PE-CF594 (BD, GL3), anti-CD24-BV605 (Biolegend, M1/69), anti- Vγ2 TCR-BV786 (BD, UC3-10A6), anti-CD23p19-Alexa Fluor 488 (Invitrogen, fc23cpg), anti-Ki-67- PerCP-eFluor 710 (Invitrogen, SolA15), anti-GL3-PE (BD, GL3), anti-IL-17F- Alexa Fluor 488 (Biolegend, 9D3.1C8), anti-Vγ1.1 TCR-BV421 (BD, 2.11), anti- Vγ1.1+ Vγ1.2 TCR-PE (Biolegend, 4B2.9), anti-Vγ3 TCR-BV510 (BD, 536), anti-CD45.1- Pacific Blue (Biolegend, A20), anti-CD3-APCCy7 (BD, 145-2C11), anti-Vδ6.3/2 TCR (BD, 8F4H7B7). 17D1 hybridoma (anti-Vγ6 antibody) and biotinylated anti-Vγ7 antibody was used as previously described (21). Cells were analyzed on an LSR (Foretessa BD Biosciences) and data were processed using FlowJo software (Tree Star).

### Tetramers and Cell Enrichment

Biotinylated PBS57 loaded CD1d monomers and 5-OP-RU loaded MR1 monomers were obtained from the tetramer facility of the US National Institutes of Health (NIH). Biotinylated monomers were tetramerized using streptavidin-phycoerythrin (PE) (Prozyme), streptavidin-allophycocyanin (APC) (Prozyme), and streptavidin-PE-Cy7 (BD). For simultaneous enrichment of NKT, MAIT, and γδ T cells, single cell suspensions of lung were stained with PBS57-CD1d PE-Cy7, 5-OP-RU-MR1 PE, and anti-TCRγδ (GL3) PE-TR and enriched with anti-PE microbeads (Miltenyi) according to the manufacturer’s instructions.

### Cell Preparation

Mice were sacrificed at the indicated time points and BAL fluids were collected in 1 mL PBS. To remove circulating cells, 15 ml of PBS was injected into the heart after incision of the abdominal aorta. Harvested lung tissues were minced by McIlwain Tissue Chopper and digested in 5 mL of RPMI-1640 containing collagenase D (400 Mandl Units; ROCHE) and DNase I (1 mg/ml; 9003-98-9) on a shaker at 37°C for 45min, followed by filtration through a 70 μm strainer and 40%, 70% Percoll (Merck) gradient centrifugation (20 min at 2,000 rpm at room temperature). To isolate LP cells from the intestine, we followed previous report (26). Single-cell suspensions were prepared and separated by Percoll gradient centrifugation. Adipose tissues were minced and digested with collagenase type IV (100 units, Gibco) and collagenase D (400 Mandl Units, ROCHE) on a shaker at 37°C for 45min. The ears were excised and cut into small pieces. The ear epithelial cell layer was removed by vigorous stirring in PBS containing 3% FBS, 20 mM HEPES, 100 U/ml penicillin, 100 μg/ml streptomycin, 1 mM sodium pyruvate, and 10 mM EDTA at 37°C for 20 min. The tissue samples were then digested in PRMI containing collagenase type V (1 mg/ml, Sigma) at 37°C for 45 min. Total cells were counted using a VI-CELL Cell Viability Analyzer (BECKMAN COULTER) and stained for FACS analysis.

### IL-1β Cytokine Measurement

For intracellular cytokine staining of proIL-1β, single cells were primed with lipopolysaccharide (LPS, 10 ng/ml; Sigma) for 2 hours in 10% FBS and 1x penicillin and streptomycin (P/S) containing RPMI media, as previously described (27, 28). Cells were co-incubated with 1x Monensin (Biolgened, 420701) and 1x Brefeldin A (Sigma). Intracellular cytokine staining was surface stained, fixed with IC fixation buffer (eBioscience), and permeabilized with the staining buffer (eBioscience).

### Immunofluorescence

Immunofluorescence staining was performed as described previously (29), with modifications. Briefly, tissues were fixed with 4% paraformaldehyde (PFA) for 1 hour and snap frozen. Five micrometer tissue sections were blocked with 5% bovine serum albumin and goat sera (Jackson Laboratory) for 1 hour at 25°C and stained with antibodies. Images were obtained using Leica DM6B with THUNDER system.

### Statistical Analysis

Prism software (GraphPad, Version 8.4.2) was used for statistical analysis, and all data were represented as mean ± SD. Unpaired two-tailed *t*-tests and one-way ANOVAs were used for data analysis and the generation of *P* values. *P* < 0.05 was considered significant.

## Results

### Peripheral Homeostasis of Tγδ17 Cells Is Dependent on Commensal Microbiome

**Figure 1:**
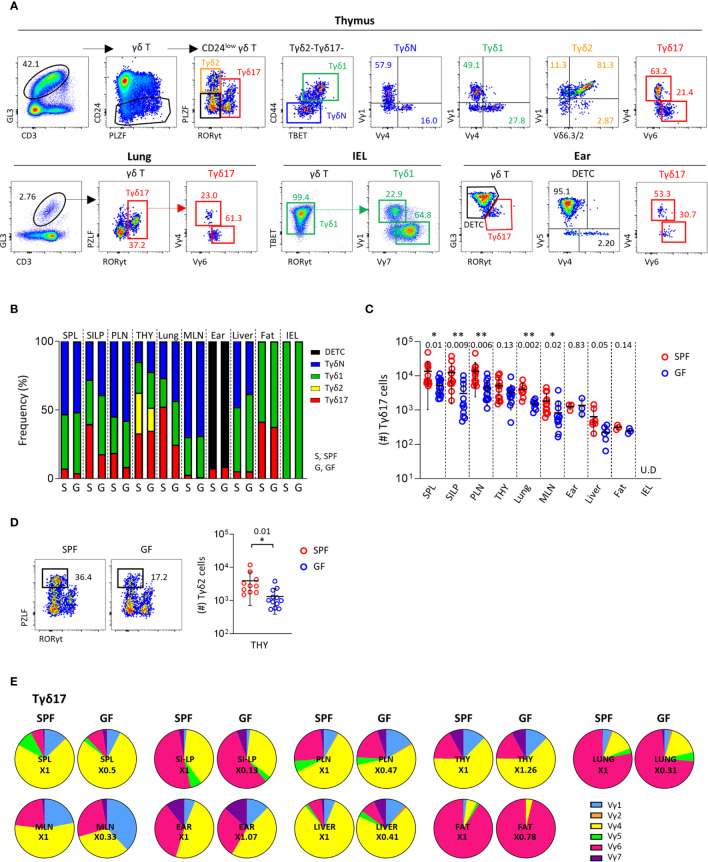
Peripheral homeostasis of Tγδ17 cells is dependent on commensal microbiome. **(A)** FACS gating strategy is shown for analysis of Vγ TCR repertoire of each subset of γδ T cells in thymus, lung, IEL and ear of B6 mice. **(B–E)** Single cell suspensions of indicated organs from SPF and GF (6-week-old) C57BL/6 (B6) mice were analyzed by flow cytometry. Thymus is gated on CD24^low^ cells. Numbers indicate frequencies of cells in adjacent gates. **(B)** Bar graphs show mean frequencies of Tγδ17, Tγδ2, Tγδ1, naïve γδ T (TγδN), and dendritic epidermal T cells (DETC) cells among total γδ T cells and CD24^low^ cells (thymus). **(C, D)** Graphs show statistical analysis of absolute numbers of Tγδ17 cells in indicated tissues **(C)** and Tγδ2 cells in the thymus **(D)**. Numbers indicate *P* values. Representative dot plots show Tγδ2 cells in the thymus. **(E)** Pie charts show mean frequencies of each Vγ TCRs among total Tγδ17 cells. Numbers indicate fold changes. Pooled data from at least three independent experiments are shown (N = 3 ~ 14). Each dot represents an individual mouse and horizontal bars show mean values. Data are presented as mean ± SD. U.D, undetected. Unpaired two-tailed *t*-test was used. *P < 0.05, **P < 0.01. SPF, specific pathogen free; GF, germ-free; SPL, Spleen; SI-LP, small intestinal lamina propria; PLN, peripheral lymph node; THY, thymus; MLN, mesenteric lymph node; IEL, intraepithelial lymphocytes.

To analyze γδ T cells systematically, we used a combination of transcription factors and surface markers as previously described (21), and newly defined naïve γδT (TγδN) cells as PLZF^lo/-^RORγt ^–^ Tbet ^–^ CD44^lo^ cells in the thymus (**Figure 1A**, upper panels). We further analyzed TCR Vγ usage using a panel of antibodies specific for TCR Vγ1, Vγ1/2, Vγ4, Vγ5, Vγ6, and Vγ7 in a single staining panel. In this way, we analyzed γδ T cells in the thymus and periphery, and phenotyped different subsets of γδ T cells with different TCR Vγ chain usages, except TCR Vγ3, which is a pseudogene.

As previously reported (30), Tγδ17 cells mainly consist of PLZF^lo^Vγ4^+^ and PLZF^int^Vγ6^+^ cells both in the thymus and periphery, including the lung and skin (**Figure 1A** and **Supplementary Figure 1**). In the thymus, all subtypes of γδ T cells were present and most intraepithelial lymphocytes (IEL) γδ T cells were TBET^+^ Tγδ1 cells. The majority of thymic TγδN and Tγδ1 cells consisted of Vγ1^+^ and Vγ4^+^ cells, whereas more than half of IEL Tγδ1 cells expressed TCR Vγ7, indicating that the Vγ TCR usage of γδ T cells varies depending on the tissue type, despite the same effector lineage. As shown in previous studies (31), in the skin, GL3^hi^ dendritic epidermal T cells (DETC) were TCR Vγ5^+^ and GL3^int^ dermal γδ T cells were RORγt^+^ Tγδ17 cells expressing TCR Vγ4 or Vγ6 (**Figure 1A**, lower right panels).

In the thymus and periphery of SPF mice, naïve and effector subsets of γδ T cells were variably distributed, except Tγδ2 cells that were exclusively present in the thymus (**Figure 1B** and **Supplementary Figure 2**). Because γδ T cells are affected by commensal microbiome (11, 32), we compared their subset distributions between SPF and GF mice (**Figure 1B** and **Supplementary Figure 2**). Notably, GF condition most prominently affected the numbers and frequencies of Tγδ17 cells in the lung and small intestinal lamina propria (siLP), in which commensal or foreign micro-organisms are abundant **(** **Figure 1C** **)**. TγδN and Tγδ1 cells were also slightly reduced in the spleen, mesenteric lymph node (mLN), and IEL of GF mice compared SPF control. Interestingly, there were decreased numbers of Tγδ2 cells, but not other subsets in thymi of GF mice **(** **Figure 1D** **)**, indicating that commensal microbiota affects thymic development of γδ T cells. We further investigated the effect of microbiome on Vγ TCR chain usage; however, there were no noticeable differences in the thymus and periphery between SPF and GF mice (**Figure 1E**, **Supplementary Figure 3** and **Supplementary Table 1**). Overall, these findings indicate that TCR Vγ repertoire determined in the thymus is not affected by commensal microbiome in the periphery, suggesting that innate signaling rather than TCR engagement by specific antigens regulates the peripheral pool of γδ T cells.

### Microbial Colonization of GF Mice Restores Peripheral Pool of Tγδ17 Cells

**Figure 2:**
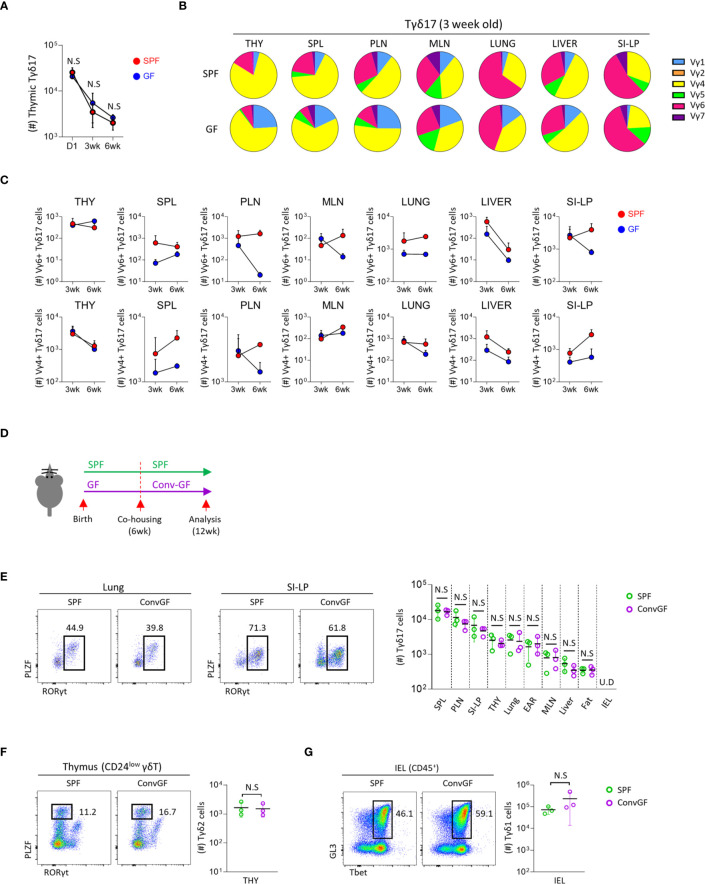
Microbial stimulation restores peripheral pool of Tγδ17 cells. Single cell suspension of indicated organs from SPF, GF and conventionalized GF B6 mice were analyzed by flow cytometry. **(A)** Graph shows statistical analysis of absolute number of thymic Tγδ17 cells at indicated ages. **(B, C)** Three and six week-old SPF and GF mice were analyzed using flow cytometry. **(B)** Pie charts show mean frequencies of each Vγ TCR among total Tγδ17 cells in indicated tissues from SPF and GF 3-week-old B6 mice (N = 3). **(C)** Graphs show statistical analysis of Vγ6^+^ and Vγ4^+^ Tγδ17 cells in indicated tissues from SPF and GF mice (N = 3). **(D–G)** GF mice were conventionalized by co-housing with SPF mice for 6 weeks (ConvGF) and analyzed. **(D)** Experimental scheme is shown. **(E)** Representative dot plots show Tγδ17 cells in the lung and SI-LP from SPF and ConvGF mice. Graph shows statistical analysis of absolute number of Tγδ17 cells indicated tissues. **(F)** Representative dot plots show thymic Tγδ2 cells and graph shows statistical analysis of their absolute numbers. **(G)** Representative dot plots show IEL Tγδ1 cells and graph shows statistical analysis of their absolute numbers. Numbers indicate frequencies of cells in adjacent gates and each dot represents an individual mouse. Error bars indicate ± SD. Pooled results from three independent experiments are shown. U.D, undetected. Unpaired two-tailed *t*-test was used. *N.S, not significant*; SPF, specific pathogen free; GF, germ-free; THY, Thymus; SPL, Spleen; PLN, peripheral lymph node; MLN, mesenteric lymph node; SI-LP, small intestinal lamina propria; IEL, intraepithelial lymphocytes.

Because maternal commensal microbiome affects the fetal immune system (33–35), we analyzed its effect on the development of Tγδ17 cells using new-born (day 1) mice, which have a fetal repertoire of γδ T cells. The numbers of thymic Tγδ17 cells were not different between SPF and GF mice at all ages **(** **Figure 2A** **)**, and there were no substantial differences in their numbers and TCR Vγ usage of thymic immature Tγδ17 (CD24^hi^ RORγt^+^) and mature Tγδ17 (CD24^lo^ RORγt^+^) cells in the neonatal mice **(** **Supplementary Figures 4A, B** **)**. In 3-week-old pre-weaned GF mice, there was increased usage of TCR Vγ1 in Tγδ17 cells compared SPF mice **(** **Figure 2B** and **Supplementary Figures 4C, D** **)**. In the periphery, Vγ4^+^ or Vγ6^+^ peripheral Tγδ17 cells were variably decreased in GF mice compared to SPF mice **(** **Figure 2C** **)**.

The development of mucosal associated invariant T (MAIT) cells is dependent on the microbiome, and later colonization of GF mice failed to reconstitute their development (36). We tested this in γδ T cells by cohousing 6-week-old GF mice with SPF mice for 6 weeks **(** **Figure 2D** **)**. However, unlike MAIT cells, in these mice, we observed that not only peripheral Tγδ17 cells, including the lung and siLP **(** **Figure 2E** **)**, but also thymic Tγδ2 **(** **Figure 2F** **)** and IEL Tγδ1 **(** **Figure 2G** **)** cells were all restored to equivalent levels of SPF mice. This features indicate that later colonization of the commensal microbiome is sufficient for the restoration of γδ T cells in adulthood.

### Tγδ17 Cells Are Tissue Resident

**Figure 3:**
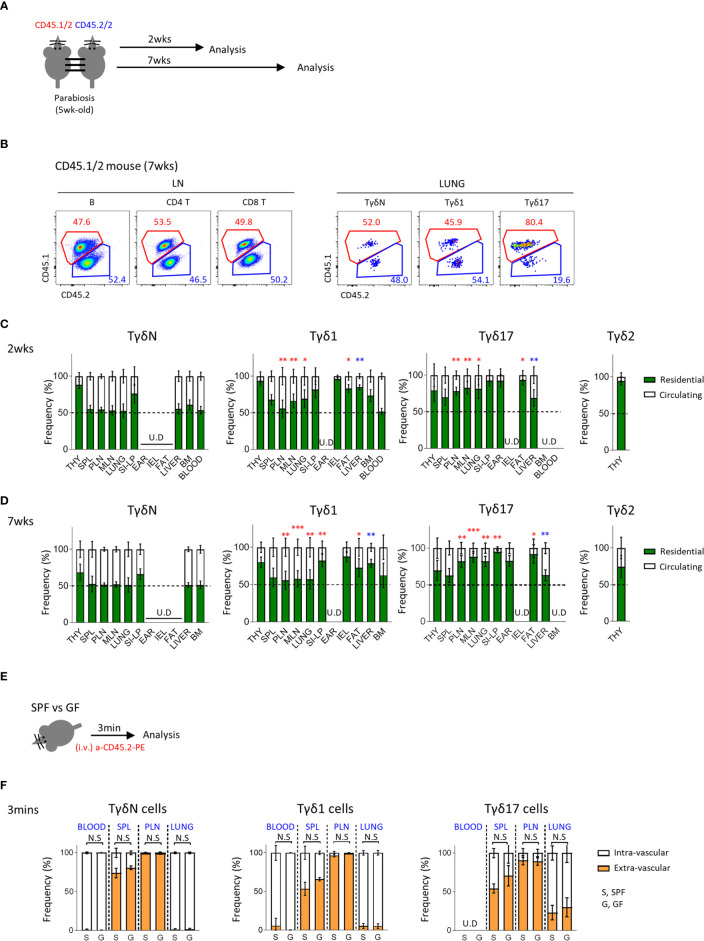
Tγδ17 cells are tissue resident. **(A)** Experimental scheme illustrates parabiosis schedules. Five week-old CD45.1/2 and CD45.2/2 congenic B6 mice were underwent parabiosis surgery and analyzed after 2- and 7- weeks. **(B)** Representative dot plots show proportion of resident (CD45.1/2) and circulating (CD45.2/2) B (B220^+^), CD4 T and CD8 T cells in peripheral lymph nodes and TγδN, Tγδ1 and Tγδ17 cells in lung. Numbers indicate frequencies of cells in adjacent gates. **(C, D)** Bar graphs show mean frequencies of residential and circulating cells of each cell subset in indicated tissues at 2- **(C)** and 7-weeks **(D)** after parabiosis. Pooled data from three independent experiments using 3 to 5 pairs are shown. **(E, F)** B6 SPF and GF mice were stained with anti-CD45.2 antibody *via* intravenously (i.v.) injection and single cell suspensions of indicated organs were analyzed at 3 min after *in vivo* staining. **(E)** Experimental scheme is shown. **(F)** Bar graphs show mean frequencies of intra- and extra- vascular cells of each cell subset in indicated tissues (N = 3). Error bars indicate ± SD. U.D, undetected. Unpaired two-tailed *t*-test was used. *P < 0.05, **P < 0.01, ***P < 0.001. THY, thymus; SPL, spleen; PLN, peripheral lymph node; MLN, mesenteric lymph node; SI-LP, small intestinal lamina propria; IEL, intraepithelial lymphocytes; BM, bone-marrow, SPF, specific pathogen free; GF, germ-free.

γδ T cells are generally known to be tissue resident. To better understand the circulating dynamics of each subset of γδ T cells in the periphery, we generated a parabiosis model using C57BL/6 congenic mice and analyzed them 2 or 7 weeks later **(** **Figure 3A** **)**. We first confirmed that 50% of B cells in the LN were from paired parabionts (**Figure 3B** and **Supplementary Figure 5A**) and analyzed γδ T cells. Interestingly, 50% of TγδN cells in most lymphoid and non-lymphoid organs, except thymus and siLP, were derived from paired parabionts, indicating that they are a circulating population similar to B cells. Tγδ17 cells were mostly tissue resident, especially in fat, ear skin, siLP, and lung. Tγδ1 cells were also tissue resident, especially in IEL and siLP. Generally, Tγδ1 cells showed a less tendency of tissue residency compared to Tγδ17 cells **(** **Figures 3C, D** **)**. As known that Vγ5^+^ DETCs are only generated during the fetal period and reside in the skin (37–39), 97% of them were tissue resident at 2 and 7 weeks after parabiosis **(** **Supplementary Figure 5** **)**. Therefore, each subset of γδ T cells has different residential or circulatory characteristics with some variability depending on the tissues they localize. Consistent with a previous report (40), we also observed that invariant natural killer T (iNKT) cells, including both NKT1 and NKT17 cells, are tissue resident **(** **Supplementary Figure 6** **)**. However, there was not much difference in their tissue residency between NKT1 and NKT2 cells at the second week of parabiosis, and there were no naïve NKT cells. Taken together, unlike previous thoughts, these findings indicate that each subset of γδ T cells has unique pattern of tissue residency, that is Tγδ17 cells are mostly resident in the peripheral tissues compared to Tγδ1 cells, and TγδN cells are circulating cells.

We additionally compared tissue residency of γδ T cells using intravascular staining of anti-CD45 antibodies (**Figures 3E, F** and **Supplementary Figure 7**) and found no significant differences between SPF and GF mice. Although intravascular staining does not necessarily differentiate tissue resident population as some cells are intravascular resident, this result suggests that the absence of microbiome does not affect circulating tendency of γδ T cells.

### Type 17 Innate T Cells Express IL-1R in the Lung

**Figure 4:**
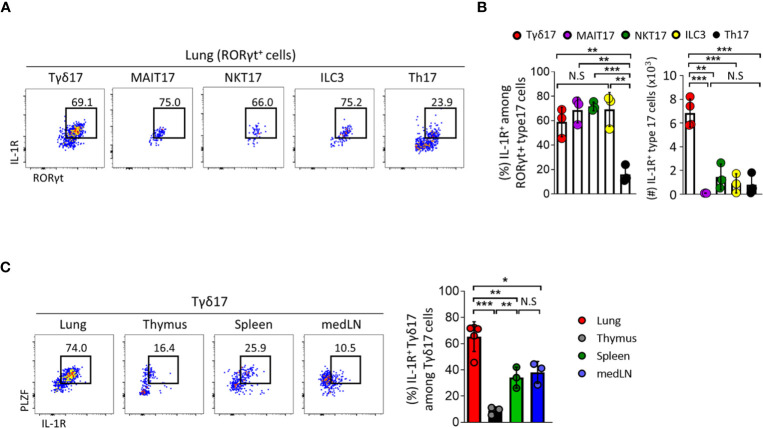
Type 17 innate T cells express IL-1R in the lung. **(A–C)** IL-1 receptor (IL-1R) expression was analyzed by flow cytometry on type 17 innate T cells, innate lymphoid cells (ILC3) and T helper (Th17) cells in indicated tissues from B6 SPF adult mice at steady state. **(A)** Representative dot plots show pulmonary IL-1R-expressing Tγδ17, mucosal associated invariant T (MAIT17), natural killer T (NKT17), ILC3, and Th17 cells. **(B)** Bar graphs show statistical analysis of frequencies and absolute numbers of **(A)**. **(C)** Representative dot plots show PLZF and IL-1R expression of Tγδ17 cells in indicated tissues. Numbers indicate frequencies of cells in adjacent gates. Data are representative of at least two independent experiments and error bars indicate ± SD. medLN, mediastinal lymph nodes. Unpaired two-tailed *t*-test and one-way ANOVA was used. N.S, not significant, *P < 0.05, **P < 0.01, ***P < 0.001.

Tγδ17 cells are activated and rapidly produce IL-17 in response to IL-1 without TCR engagement (32, 41, 42). In the lung, γδ T cells express copious amounts of IL-1R and their over activation due to excessive IL-1 leads to poor control of lung adenocarcinoma (8, 43). Based on this, we further analyzed the expression pattern of IL-1R in γδ T cells and compared it with those in other types of innate T cells such as NKT, MAIT, conventional CD4 T cells, and innate lymphoid cells (ILCs) (**Figures 4A, B** and **Supplementary Figure 8**). Frequencies of IL-1R expression in type 17 innate T cells including Tγδ17, NKT17, and MAIT17 cells and in ILC3s were comparable with one another at approximately 60–70%, whereas only about 20% of conventional Th17 cells expressed IL-1R. However, the number of IL-1R-expressing cells was highest in Tγδ17 cells occupying 68% **(** **Figure 4B** **)**. In addition, pulmonary Tγδ17 cells expressed the highest level of IL-1R compared to those in thymus, spleen, and mediastinal lymph node (medLN) **(** **Figure 4C** **)**. Together, these findings suggest that Tγδ17 cells would be the main population that responds to exogenous IL-1 and produces IL-17.

### PM Induces IL-1β Secretion and Acute Neutrophilia *via* Tγδ17 Cells

**Figure 5:**
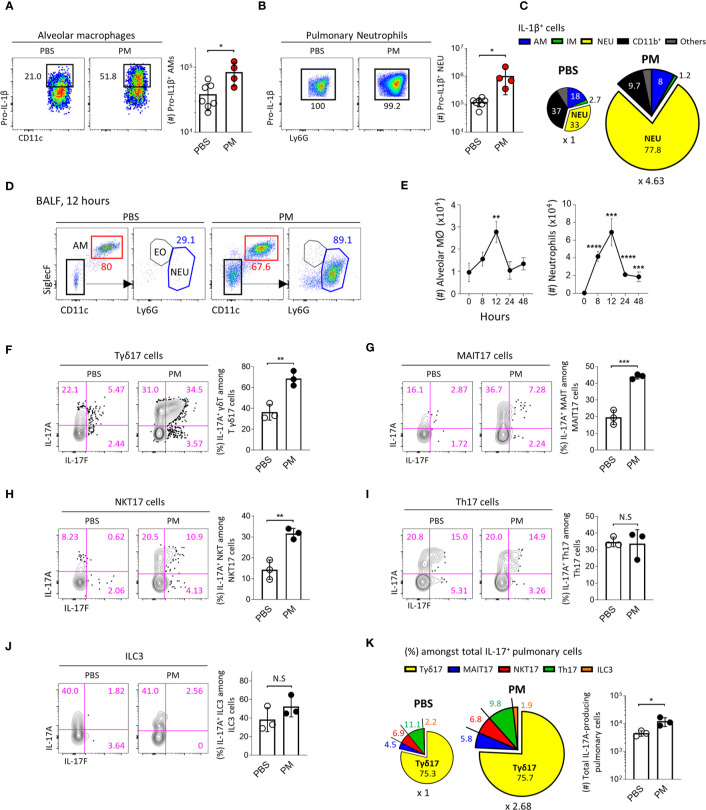
PM induces IL-1β secretion and acute neutrophilia *via* Tγδ17 cells. **(A–C)** B6 mice were intranasally administered with 250 μg of PM or PBS and single cell suspensions of lung tissue were analyzed at 4 hours after PM exposure. **(A, B)** Representative dot plots show alveolar macrophages **(A)** and neutrophils **(B)**. Bar graphs show statistical analysis of absolute number of pro-IL-1β-producing cells. **(C)** Pie charts show mean frequencies (proportional to angle) and numbers (proportional to area) of indicated cells among total pro-IL-1β-producing cells. **(D, E)** B6 mice were intranasally administered with 250 μg of PM and analyzed at indicated time points. **(D)** Representative dot plots show alveolar macrophages (AM), neutrophils (NEU) and eosinophils (EO) in broncho-alveolar lavage fluid (BALF) harvested at 12 hours after PM administration. **(E)** Graphs show the numbers of alveolar macrophages and neutrophils at indicated time periods in BALF (N = 2 ~ 4). **(F–K)** B6 mice were intranasally administered with 250 μg of PM and mononuclear cells of lung tissue were analyzed at 24 hours after PM exposure. Representative contour plots show IL-17A^+^ or IL-17F^+^ pulmonary Tγδ17 **(F)** MAIT17 **(G)** NKT17 **(H)** Th17 **(I)** and ILC3 cells **(J)**. Bar graph shows statistical analysis of frequencies of IL-17A-producing cells. **(K)** Pie charts show mean frequencies (proportional to angle) and numbers (proportional to area) of indicated cells among total IL-17A-producing pulmonary CD45^+^ cells. Numbers indicate frequencies of cells in adjacent gates or frequencies in each area **(C, K)**. Each dot represents an individual mouse and error bars indicate ± SD. Unpaired two-tailed *t*-test was used. N.S, not significant, *P <0.05, **P < 0.01, ***P < 0.001, ****P < 0.0001. AM, alveolar macrophage; IM, interstitial macrophage; NEU, neutrophils; EO, eosinophil.

To investigate the pathogenic role of IL-1R^+^ lung-resident Tγδ17 cells *in vivo*, we used a mouse model of PM-induced acute airway inflammation. We intranasally administered mice with 250 μg of PM and analyzed at each time points after exposure. Lung epithelial cells are known to produce IL-1β in response to PM (14) and we further analyzed CD45^+^ leukocytes by flow cytometry. The mean fluorescence of intensity of IL-1β was sharply increased, as well as, the number of IL-1β-producing cells was increased after PM exposure **(** **Supplementary Figure 9A** **)**. We analyzed the intracellular IL-1β in the CD45^+^ leukocytes using a gating strategy as depicted in **Supplementary Figure 9B** to include T cells, B cells, AM, interstitial macrophages (IM), neutrophils, and other undefined CD11b^+^ cells. After 4 hours of PM exposure, AM and neutrophils remarkably produced IL-1β **(** **Figures 5A, B** **)**. Interestingly, the major cellular sources of IL-1β were neutrophils (33%) and CD11b^+^ cells (37%) in normal lungs, and neutrophils produced most of IL-1β (approximately 80%) in PM-exposed lungs **(** **Figure 5C** **)**. Consistent with previous reports (18, 19), we found that PM causes an acute expansion of alveolar macrophages and strong neutrophilia in the airways **(** **Figure 5D** **)**. The kinetics of neutrophil influx was similar to that of alveolar macrophages and peaked at 12 hours after PM administration **(** **Figure 5E** **)**.

Since IL-17-producing Tγδ17 cells are associated with neutrophilia in the lung after bacterial or viral infection (44, 45), we next examined whether PM induces the production of IL-17 from γδ T cells. Using a gating strategy as shown in **Supplementary Figure 10**, we found that the frequencies of IL-17-producing Tγδ17, MAIT17, and NKT17 cells significantly increased **(** **Figures 5F–H** **)**, whereas there were no changes in IL-17 production from Th17 cells and ILC3s **(** **Figures 5I, J** **)** 24 hours after PM exposure. We also confirmed that the total number of IL-17-producing cells was approximately 2.68 times higher in PM-treated lungs compared to that in PBS-treated group (**Figure 5K**, pie charts). Notably, we discovered that Tγδ17 cells produce 75% of IL-17 under both normal and inflammatory conditions **(** **Figure 5K** **)**. PM exposure not only enhanced IL-17 production from Tγδ17 cells **(** **Figure 5F** **)**, but also expanded their numbers upon its consecutive exposure for 4 days **(** **Supplementary Figures 11A, B** **)**. However, numbers of Tγδ1 or TγδN cells were not increased and there were rather decreased IFNγ secretion from Tγδ1 cells **(** **Supplementary Figures 11C, D** **)**. Taken together, these findings indicate that innate T cells, but not Th17 CD4 T cells or ILC3s, are the source of early IL-17 upon PM exposure.

### Commensal Microbiota Promotes PM-Induced Acute Neutrophilic Airway Inflammation

**Figure 6:**
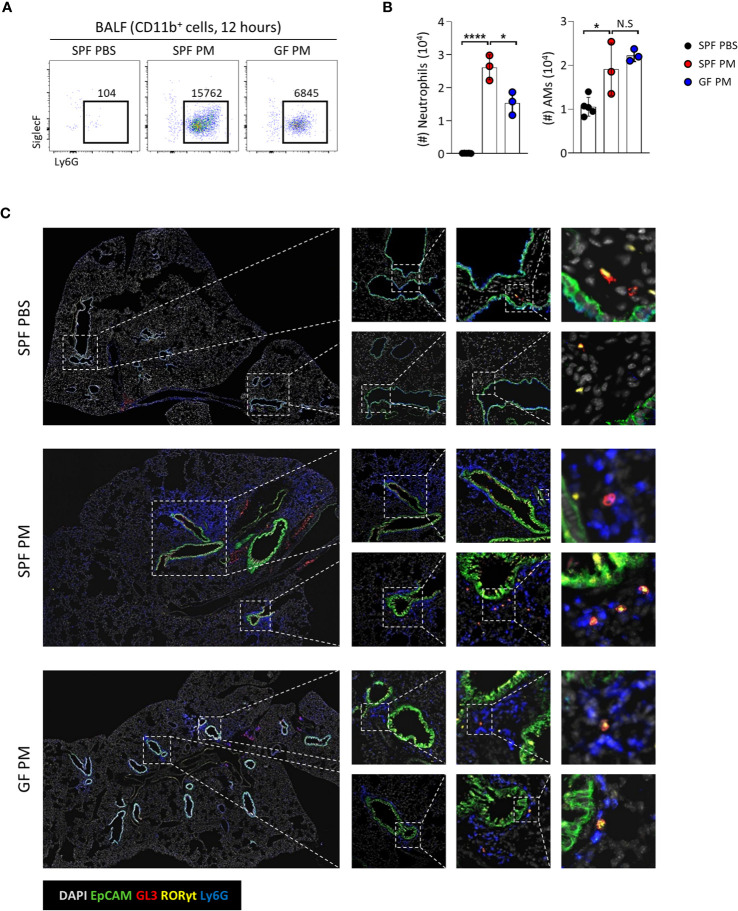
Commensal microbiota promote PM-induced acute neutrophilic airway inflammation. B6 SPF and GF mice were intranasally administered with 250 μg of PM or PBS and analyzed at 12 hours after PM exposure. **(A)** Representative dot plots show neutrophils in broncho-alveolar lavage fluid (BALF). Numbers indicate absolute numbers of cells in adjacent gates. **(B)** Graph shows statistical analysis of absolute number of neutrophils and alveolar macrophages (AMs) in BALF. **(C)** Representative immunohistochemical staining images of lungs from SPF PBS, SPF PM, and GF PM mouse group (N = 3 ~ 5). Each dot represents an individual mouse and horizontal bars show mean values. Data are presented as mean ± SD. Unpaired two-tailed *t*-test was used. *N.S, not significant*; *P < 0.05, ****P < 0.0001. SPF, specific pathogen free; GF, germ-free; PM, particulate matter; AMs, alveolar macrophages.

We showed that homeostasis of Tγδ17 cells is dependent on commensal microbiomes **(** **Figures 1**, **2** **)**, and PM induces acute neutrophilia with Tγδ17 expansion **(** **Figure 5** **)**. Therefore, we tested whether GF mice have reduced neutrophilic inflammation upon PM exposure **(** **Figures 6A, B** **)**. Indeed, GF mice had reduced infiltration of neutrophils and AMs upon PM exposure. Immunofluorescence staining of the lungs revealed that neutrophils clustered together with Tγδ17 cells around airways in SPF mice and GF mice had less infiltration of these cells **(** **Figure 6C** **)**. Taken together, these results show that commensal microbiomes regulate neutrophilic inflammation upon PM exposure, which is likely to be mediated by Tγδ17 cells.

### Tγδ17 Cells Promote PM-Induced Acute Pulmonary Neutrophilic Inflammation

**Figure 7:**
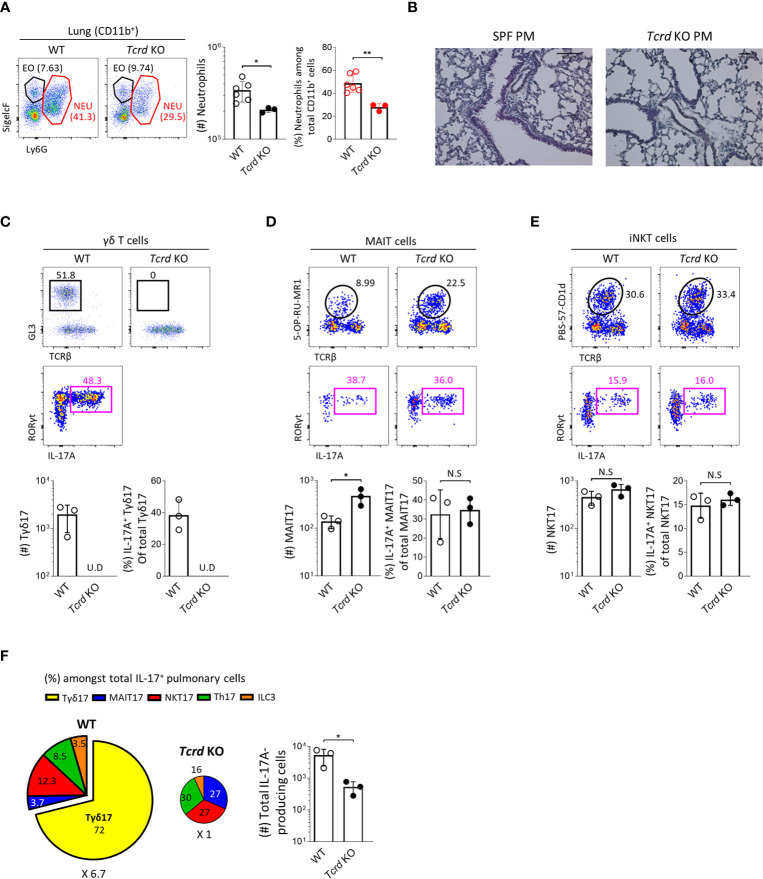
Tγδ17 cells promote PM-induced acute pulmonary neutrophilic inflammation. **(A, C–F)** B6 WT and *Tcrd* KO mice were intranasally (i.n.) administered with 250 μg of PM and single cell suspensions of lung tissue were analyzed at 24 hours after PM exposure. **(A)** Representative dot plots are shown after gating CD11b^+^ cells. Bar graph shows statistical analysis of absolute numbers of neutrophils and their frequencies among total CD11b^+^ cells. **(B)** Mice were (i.n.) administered with 250 μg of and analyzed at 12 hours after PM exposure. Representative hematoxylin-eosin (H&E) stained lung sections are shown (original magnification X200). **(C–E)** Representative dot plots show total γδ T **(C)**, MAIT **(D)** and NKT **(E)** cells (upper panels) in WT and *Tcrd* KO mice and their IL-17A production (lower panels). Bar graphs show statistical analysis of absolute numbers and frequencies of each IL-17-producing innate T cells. **(F)** Pie charts show mean frequencies (proportional to angle) and numbers (proportional to area) of indicated cells among total IL-17A-producing pulmonary cells. Bar graphs show statistical analysis of absolute numbers of total IL-17-producing cells. Numbers indicate frequencies of cells in adjacent gates **(A, C–E)** or area **(F)**. Each dot represents an individual mouse and error bars indicate ± SD. U.D, undetected. Unpaired two-tailed *t*-test was used. N.S, not significant, *P < 0.05, **P < 0.01. EO, eosinophil; NEU, neutrophil.

To obtain direct evidence that Tγδ17 cells are associated with the pathogenesis of PM-induced airway inflammation, we investigated and compared the severity of neutrophilic inflammation between B6 wild-type (WT) and TCRδ-deficient (*Tcrd ^-/-^*) mice 24 hours after PM administration. We found that *Tcrd ^-/-^* mice showed significantly decreased neutrophilia **(** **Figures 7A, B** **)** without affecting the frequencies of IL-17-producing MAIT and iNKT cells compared to those of WT mice **(** **Figures 7C–E** **)**. We and others have previously shown that MAIT cells expand in the absence of NKT or γδ T cells in the thymus and skin (21, 36). Consistent with these findings, the number of MAIT17 cells increased three times in lung of *Tcrd ^-/-^* mice. However, they could not compensate the absence of γδ T cells and there was an average 6.7-fold reduction of IL-17-producing cells in the lung after PM exposure **(** **Figure 7F** **)**. We also confirmed that neutrophilic inflammation was significantly relieved in *Il17a/f*-double knockout mice **(** **Supplementary Figure 12** **)**. Collectively, these findings indicate that Tγδ17 cells play a major role in acute neutrophilia induced by PM exposure.

**Figure 8:**
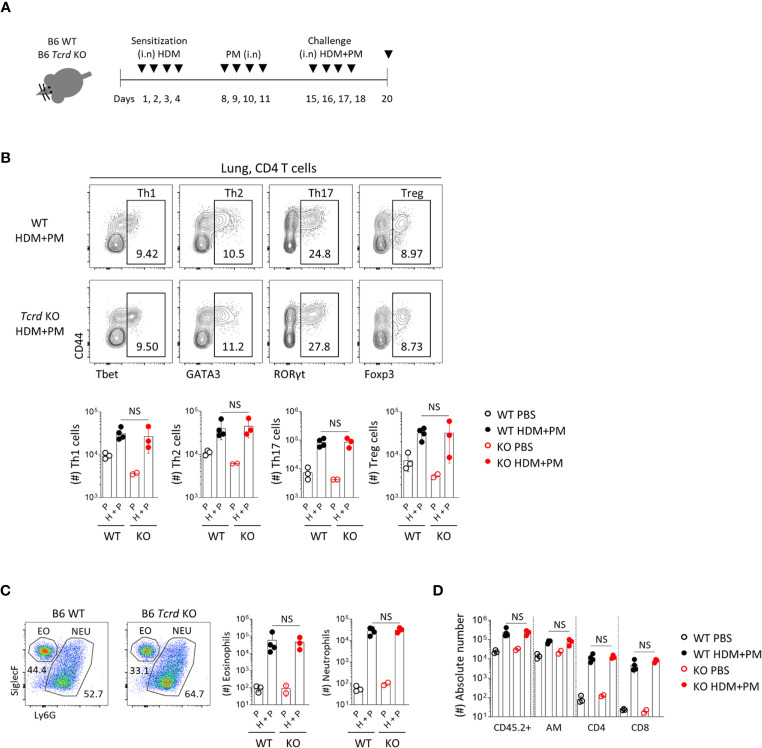
T helper 17 cells function as the main effector cells in PM-induced chronic pulmonary inflammatory condition. B6 WT and *Tcrd ^-/-^* mice were intranasally sensitized and challenged with 20 μg of HDM (*Dermatophagoides pteronyssinus*). Mice were administered with 250 μg of PM at day 8-11 and 15-18, and sacrificed at day 20. **(A)** Experimental scheme of HDM/PM-induced chronic allergic asthma is shown. **(B)** Representative contour plots show pulmonary CD4^+^ T cells. Bar graphs show statistical analysis of their absolute numbers. **(C)** Representative dot plots show eosinophils and neutrophils in BALF harvested at day 20. Bar graphs show statistical analysis of absolute numbers of eosinophils and neutrophils. **(D)** Bar graph shows statistical analysis of absolute numbers of total CD45.2^+^, alveolar macrophages, CD4 and CD8 T cells in BALF at day 20. Numbers indicate frequencies of cells in adjacent gates. Each dot represents an individual mouse and error bars indicate ± SD. Unpaired two-tailed *t*-test was used. *N.S, not significant*; EO, eosinophil; NEU, neutrophil; AM, alveolar macrophage.

We further analyzed the effect of γδ T cells in a chronic allergic asthma model induced by HDM and PM **(** **Figure 8A** **)**. Previous reports showed that diesel dust converted allergic asthma from a Th2 to Th17-dominant inflammatory model (18, 19), and we also found that co-administration of HDM and PM induced the dominant expansion of RORγt^+^ CD4 T cells **(** **Figure 8B** **)**. In *Tcrd ^-/-^* mice, however, there was no decreased infiltration of neutrophils or other immune cells **(** **Figures 8C, D** **)**, indicating that γδ T cells do not influence the chronic model of Th17-dominant inflammation.

## Discussion

In this study, we found that the commensal microbiome mainly regulates the peripheral homeostasis of Tγδ17 and thymic Tγδ2 cells. We categorized γδ T cells in the thymus and peripheral tissues according to transcription factors and surface marker expression, as TγδN, Tγδ1, Tγδ2, and Tγδ17 cells. By using 6 different anti-TCRγ antibodies, we analyzed TCRγ usage in each subset and compared them between SPF and GF mice. In 3-week-old GF mice, we found that the proportion of Vγ1 usage increased whereas Vγ6 usage decreased **(** **Figure 2B** **)**. Unlike the previous notion that γδ T cells reside in tissues, we found that γδ T cells have different residential/circulating phenotypes for each subset and their localization. In particular, TγδN cells exhibit a circulating phenotype, while Tγδ1 and Tγδ17 cells reside in tissues, especially in lungs and siLP, where they constantly encounter environmental components and microbial antigens. These results suggest that tissue-resident Tγδ17 cells can rapidly induce an immune response in inflammatory conditions.

Given the importance of IL-1/IL-1 receptor (IL-1R) signaling in the activation of Tγδ17 cells (32, 41, 42), we showed that type 17 innate T cells and ILC3s express higher levels of IL-1R than conventional CD4 T cells in the lung **(** **Figures 4A, B** **)**. Among IL-1R-expressing cells, pulmonary Tγδ17 cells were the majority and expressed higher levels of IL-1R than those in other tissues **(** **Figure 4C** **)**. These features suggest that Tγδ17 cells produce IL-17 most effectively in response to IL-1 signaling in lungs compared to those in other tissues. To define the pathological role of pulmonary Tγδ17 cells, we used a mouse model of PM-induced acute airway inflammation and HDM/PM-induced chronic allergic asthma. As previously described (18, 19), we showed that PM significantly aggravate neutrophilic inflammation in the airways and induce the production of IL-1β **(** **Figures 5A–E** **)** signaling to lung-resident IL-1R^+^ Tγδ17 cells. In mice deficient for γδ T cells (*Tcrd ^-/-^* mice) and IL-17 (*Il17a/f ^-/-^* mice), acute neutrophilic inflammation was significantly relieved (**Figure 7** and **Supplementary Figure 12**). However, there were no noticeable differences in allergic immune responses between WT and *Tcrd ^-/-^* mice under chronic allergic conditions **(** **Figure 8** **)**. We speculate that this might be due to the efficient development of Th17 CD4 T cells that replace the requirement of Tγδ17 cells in the chronic phase.

Previous report showed that TLR ligands driven from microbiome can stimulate the production of IL-1β, leading to proliferation and activation of lung-resident γδ T cells thereby further augment inflammatory responses (8). Other studies also suggested that commensal microbiomes are required to maintain IL-1R1^+^ Tγδ17 cells (11, 32). Thus, these findings suggest that the commensal microbiota can orchestrate the maintenance of peripheral γδ T cells by stimulating TLR ligands and IL-1β production.

Although the relationship between the commensal microbiome and immune system has been extensively studied, there are only a few studies on the effect of microbiota on the development of γδ T cells (6, 7). Nonetheless, the use of multiple Tγδ subsets with microbiome-related variations has not been addressed. Here, we identified that even though the commensal microbiota regulates the development of γδ T cells, there are not much different Vγ TCR repertoires between SPF and GF mice, except for the 3-week-old GF mice. We have previously observed that Vγ1^+^ cells expand in SPF Vγ4/6 KO mice with undefined mechanism (21). Based on this, we speculate that the expanded Vγ1^+^ cells in 3-week-old GF mice might be due to the defective development of Tγδ17 cells with TCR Vγ4 or Vγ6. However, further investigation is required to define the molecular and cellular mechanisms of Vγ TCR plasticity.

We unexpectedly found that intestinal TγδN and Tγδ1 cells have unique properties that they have fewer Vγ1^+^ cells compared to those of other tissues **(** **Supplementary Figures 3A, B** **)**. In addition, TγδN cells in only siLP showed tissue-resident property **(** **Figures 3C, D** **)**, suggesting that specialized gut environments, such as microbial community or metabolite dynamics, might influence their tissue residency. Interestingly, we found that the Vγ TCR usage of TγδN exhibited similar patterns to that of peripheral Tγδ1 cells, which is mainly composed of Vγ1^+^ and Vγ4^+^ cells except in siLP **(** **Supplementary Figure 3A–B** **)**. These findings suggest the possibility that circulating TγδN cells differentiate into Tγδ1 cells in the tissue.

Unlike MAIT cells, we showed that later exposure of microbial stimulation is sufficient for peripheral expansion and maintenance of γδ T cells. Thymic development of γδ T cells, except Tγδ2 cells, was not affected by the microbiota, whereas mature MAIT cells are absent in the GF thymus (36, 46). It is possible that there is a specific time window for thymic development of MAIT cells and later colonization is not sufficient to restore it. In contrast, iNKT cells were not affected at all in the thymus and periphery of GF mice (47, 48), suggesting that innate T cells recognize different types of antigens for their thymic development and peripheral expansion, which requires further investigation. Unlike previously report (11), we observed only marginal difference of hepatic Tγδ17 cells between SPF and GF mice (*P* = 0.051). Since hepatic γδ T cells are dependent on gut microbiota, we speculate that this difference is due to the different gut microbiomes of different animal facilities.

In this study, we used ERM-CZ-100 and ERM-CZ120 as clinically relevant air pollutants (Sigma, PM10-like, i.e., < 10 um median aerodynamic diameter). Although the composition of PM varies from source to source, our study is consistent with previous reports on PM-induced neutrophilic inflammation (13, 18, 19). Here we show that PM induces acute airway inflammation by recruiting IL-1β-producing neutrophils, which activate IL-17-producing IL-1R^+^ Tγδ17 cells. This is consistent with previous report (49) and we speculate that Tγδ17 cells secrete IL-17, which recruits additional neutrophils to the site of inflammation, thus providing more IL-1β by feed-forward circuit.

In conclusion, our study has identified a crosstalk between the commensal microbiota and lung-resident Tγδ17 cells, and provided a mechanistic insight into PM-induced acute neutrophilia. These findings suggest that targeting γδ T cells could be a new therapeutic strategy for acute lung injury dominated by neutrophilic inflammation.

## Data Availability Statement

The raw data supporting the conclusions of this article will be made available by the authors, without undue reservation, to any qualified researcher.

## Ethics Statement

The animal study was reviewed and approved by Institutional Animal Care and Use Committee of POSTECH.

## Author Contributions

CY designed and performed experiments. D-IK performed parabiosis surgery. MK performed immunofluorescence. S-HI provided research interpretation. CY and YL analyzed data and wrote the manuscript. YL conceptualized the research. All authors contributed to the article and approved the submitted version.

## Funding

This work was supported by the National Research Foundation of Korea NRF-2019R1F1A1059237 (to YL) and the Korea Global PhD Fellowship Program (KGPF) NRF-2016H1A2A1908163 (to CY) funded by the Korean Ministry of Science Information/Communication Technology.

## Conflict of Interest

S-HI is the CEO of the ImmunoBiome.

The remaining authors declare that the research was conducted in the absence of any commercial or financial relationships that could be construed as a potential conflict of interest.
