# Supplementary material for: Commensal Microbiome Expands Tγδ17 Cells in the Lung and Promotes Particulate Matter-Induced Acute Neutrophilia
Source: Front Immunol. 2021 Mar 29;12:645741. doi: 10.3389/fimmu.2021.645741 (PMC8039457; doi:10.3389/fimmu.2021.645741)
Supplement: Supplementary file 1 [file DataSheet_1.pdf]

Supplemental Information

Commensal microbiome expands Tγδ17 cells in the lung and promotes particulate matter-induced acute neutrophilia

Chorong Yang, Dong-il Kwon, Mingyu Kim, Sin-Hyeog Im, You Jeong Lee

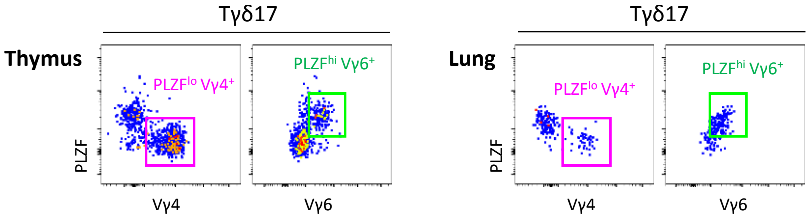

Supplementary Figure 1. Tγδ17 cells are composed of Vγ4$^+$ and Vγ6$^+$ TCR depending on the PLZF expression level. Representative dot plots show PLZF^{lo} Vγ4$^+$ and PLZF^{hi} Vγ6$^+$ Tγδ17 cells in the thymus and lung.

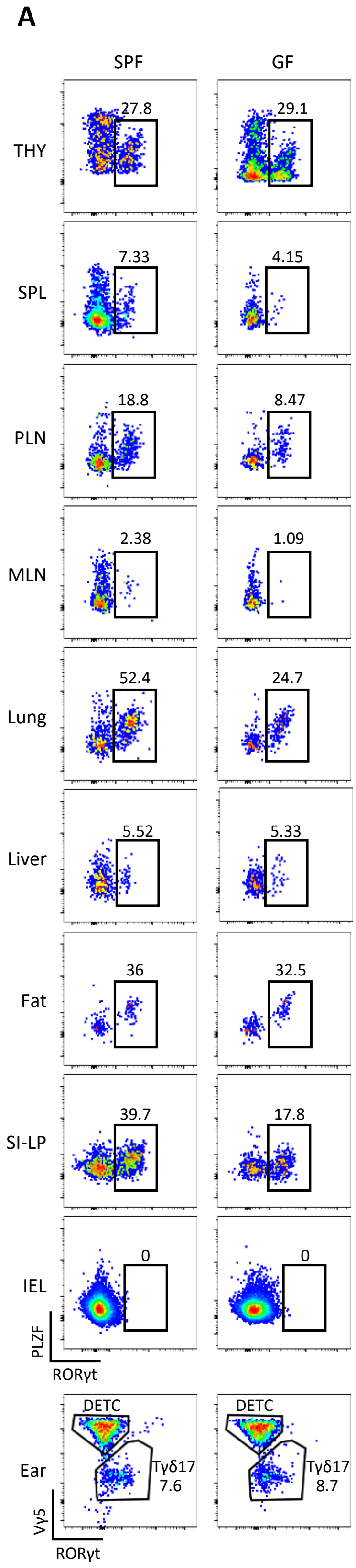

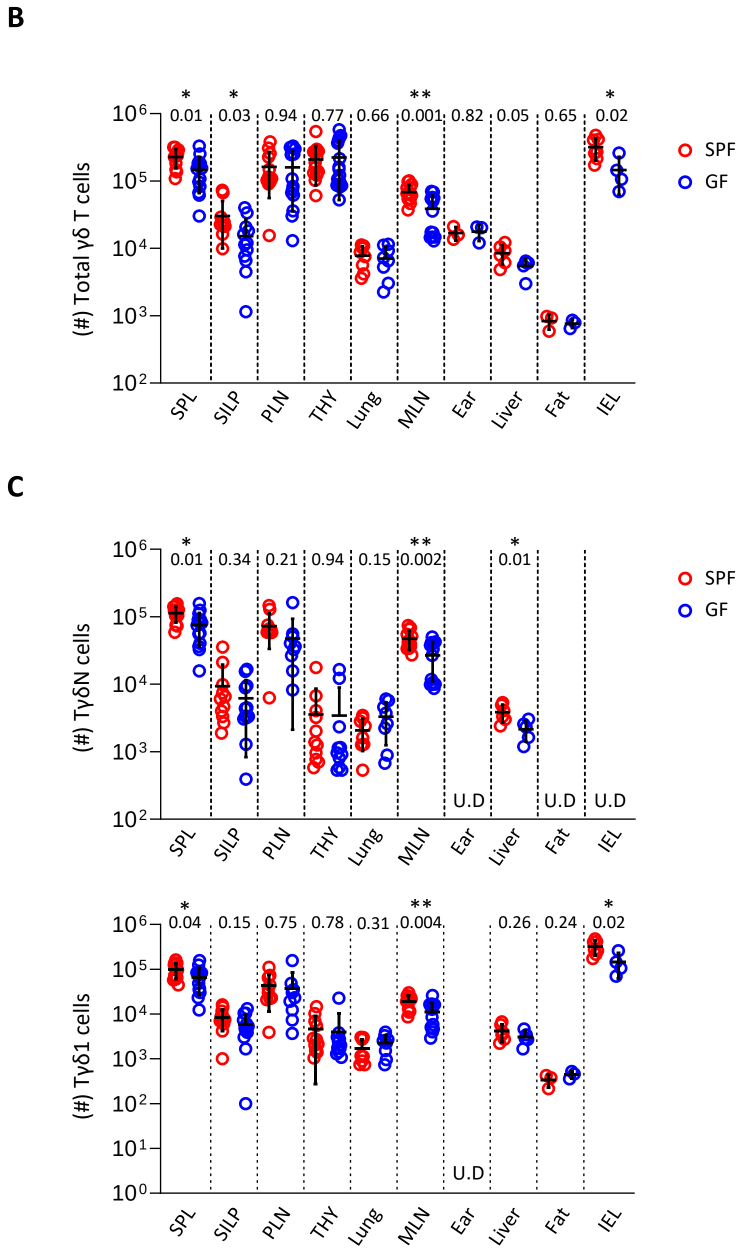

Supplementary Figure 2. Peripheral homeostasis of Tγδ17 cells is dependent on commensal microbiome. Single cell suspensions of indicated organs from SPF and GF (6-week-old) C57BL/6 (B6) mice were analyzed by flow cytometry. Thymus are gated on $CD24^{low}$ cells. **(A)** Representative dot plots show Tγδ17 cells among total γδ T cells and $CD24^{low}$ cells (thymus). Numbers indicate frequencies of cells in adjacent gates. **(B)** Graph shows statistical analysis of absolute number of total γδ T cells in indicated tissues. **(C)** Graphs show statistical analysis of absolute numbers of TγδN (upper panels) and Tγδ1 cells (lower panels) in indicated tissue. Numbers indicate *P* values. Pooled data from at least three independent experiments are shown (N=3-14). Each dot represents an individual mouse and horizontal bars show mean values. Data are presented as mean ± SD. U.D, undetected. Unpaired two-tailed *t*-test was used. **P* <0.05, ***P* <0.01. SPF, specific pathogen free; GF, germ-free; SPL, Spleen; SILP, small intestinal lamina propria; PLN, peripheral lymph node; THY, thymus; MLN, mesenteric lymph node; IEL, intraepithelial lymphocytes; DETC, dendritic epidermal T cells.

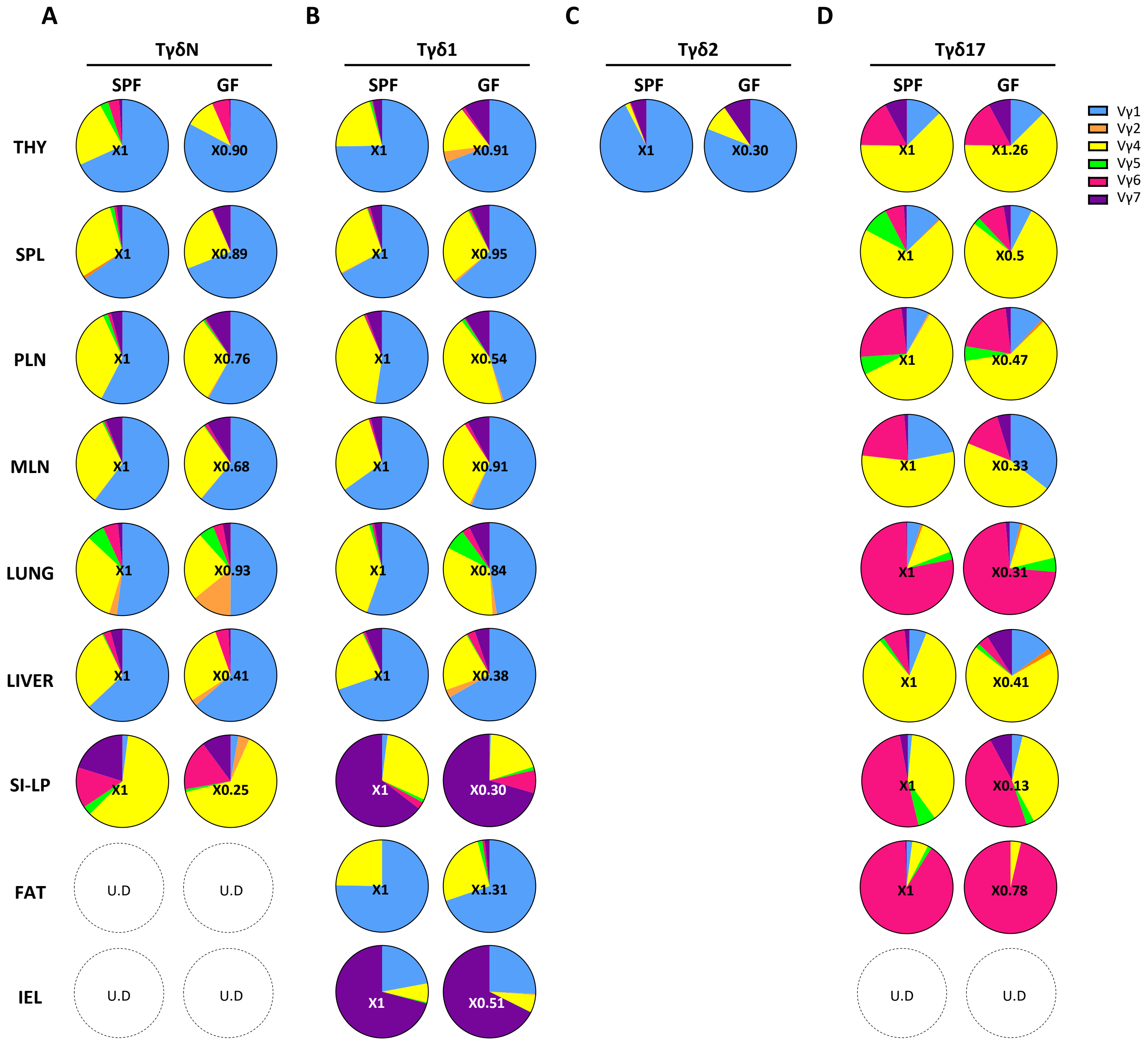

Supplementary Figure 3. Peripheral homeostasis of Tγδ17 cells is dependent on commensal microbiome. Vγ TCR repertoire of SPF and GF (6-week-old) B6 mice were analyzed in each tissue by flow cytometry. Pie charts show mean frequencies of each subset among TγδN (A), Tγδ1 (B), Tγδ2 (C), and Tγδ17 cells (D) in indicated tissues (N=3). Numbers indicate fold changes. Results from three independent sets of experiments are shown. U.D, undetected. SPF, specific pathogen free; GF, germ-free; THY, thymus; SPL, Spleen; PLN, peripheral lymph node; MLN, mesenteric lymph node; SI-LP, small intestinal lamina propria; IEL, intraepithelial lymphocytes.

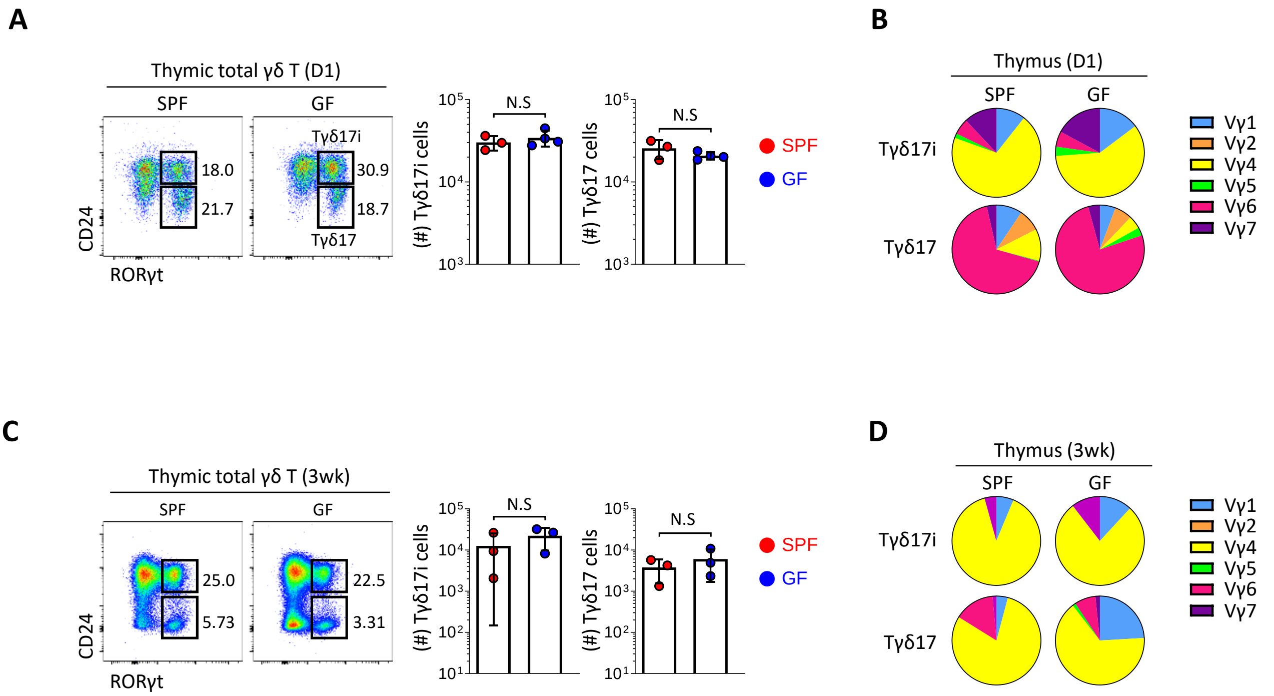

Supplementary Figure 4. Microbial stimulation restores peripheral pool of Tγδ17 cells. B6 1-day (D1), 3-week-old SPF and GF mice were analyzed using flow cytometry. **(A)** Representative dot plots show CD24^{hi}RORγt^{+} immature Tγδ17 (Tγδ17i) and CD24^{low}RORγt^{+} Tγδ17 cells in the thymus from newborn mice (1-day). Bar graphs show statistical analysis of their absolute numbers. **(B)** Pie charts show mean frequencies of each Vγ TCR subset among Tγδ17i and Tγδ17 cells (N=3-4). **(C)** Representative dot plots show thymic Tγδ17i and Tγδ17 cells and bar graph shows statistical analysis of their absolute numbers from 3-week-old mice. **(D)** Pie charts show mean frequencies of each Vγ TCR subset among Tγδ17i and Tγδ17 cells (N=3). Results from three independent sets of experiments are shown. Error bars indicate ± SD. Unpaired two-tailed *t*-test was used. *NS: not significant*.

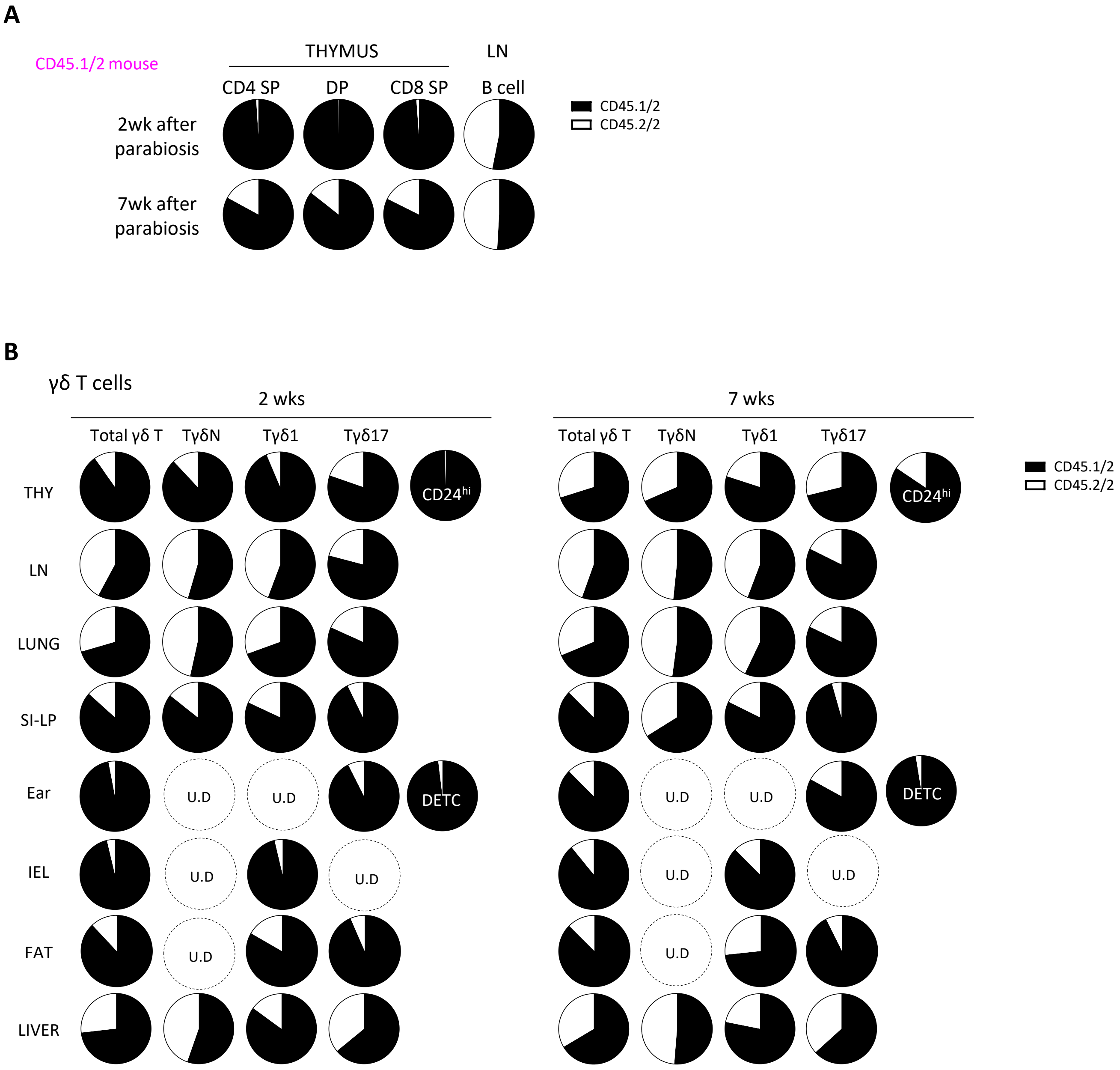

Supplementary Figure 5. Tγδ17 cells are tissue resident. B6 CD45.1/2 and CD45.2/2 congenic mice were underwent parabiosis surgery and analyzed after 2- and 7-weeks. **(A)** Pie charts show mean frequency of residential (CD45.1/2) and circulating (CD45.2/2) thymic CD4 and CD8 SP, DP thymocytes and B cells in peripheral lymph nodes at 2- and 7-weeks after surgery. **(B)** Pie charts show mean frequency of residential (CD45.1/2) and circulating (CD45.2/2) γδ T cells in indicated organs at 2-weeks and 7-weeks after surgery. Pooled results from three independent sets of experiments using 3 to 5 pairs are shown. U.D, undetected. THY, thymus; LN, lymph node; SI-LP, small intestinal lamina propria; IEL, intraepithelial lymphocytes; DETC, dendritic epidermal T cells.

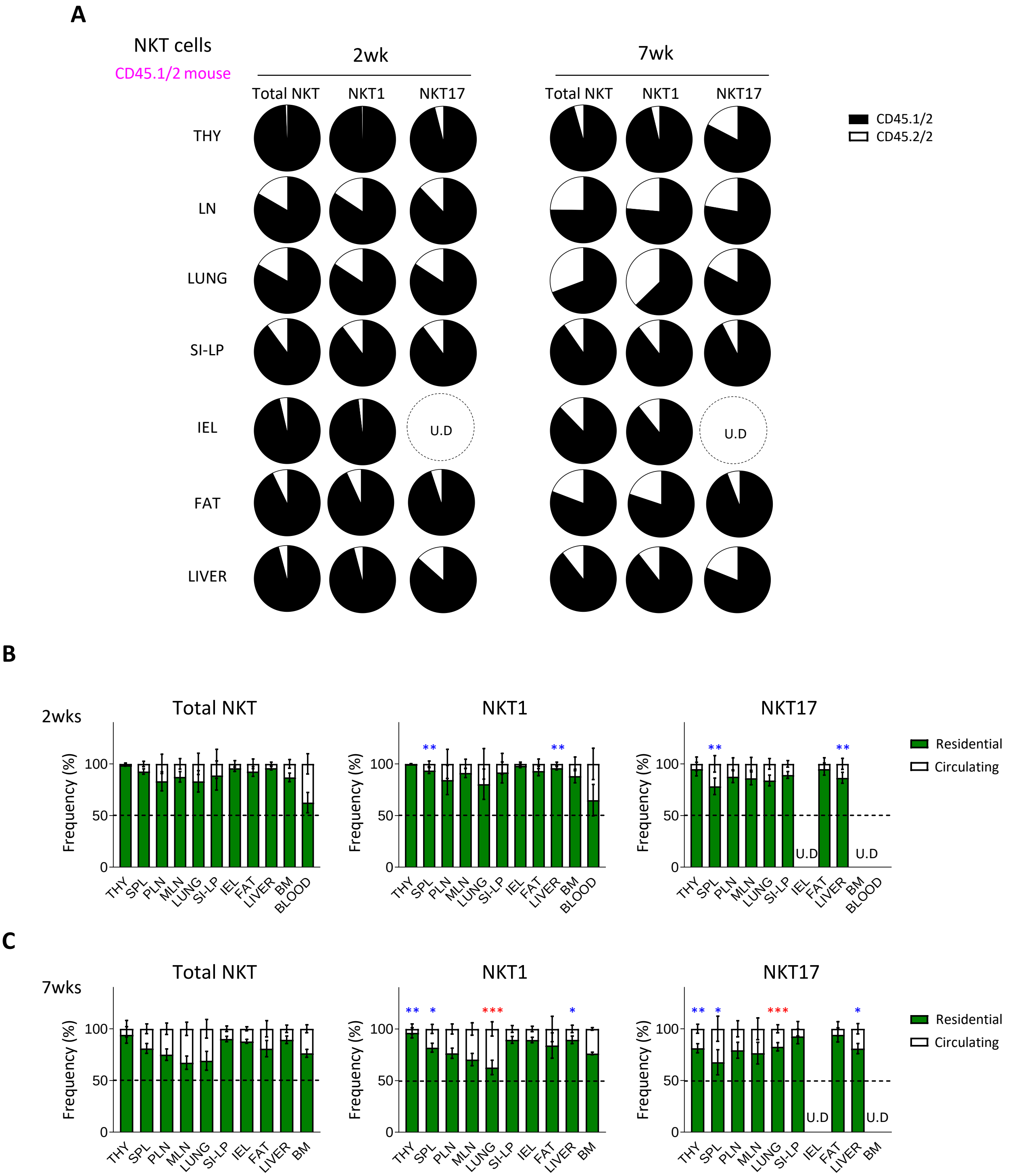

Supplementary Figure 6. NKT1 and NKT17 cells are tissue resident. B6 CD45.1/2 and CD45.2/2 congenic mice were underwent parabiosis surgery and analyzed after 2- and 7-weeks. **(A)** Pie charts show mean frequency of residential (CD45.1/2) and circulating (CD45.2/2) total NKT, NKT1, and NKT17 cells in indicated tissues at 2- and 7-weeks after surgery. **(B-C)** Bar graphs show mean frequencies of residential and circulating cells of each subset of NKT cells in indicated tissues at 2- (B) and 7-weeks (C) after parabiosis. Pooled results from three independent sets of experiments using 3 to 5 pairs are shown. Error bars indicate ± SD. U.D, undetected. Unpaired two-tailed *t*-test was used. **P* <0.05, ***P* <0.01, ****P* <0.001. THY, thymus; LN, lymph node; SI-LP, small intestinal lamina propria; IEL, intraepithelial lymphocytes; BM, bone-marrow.

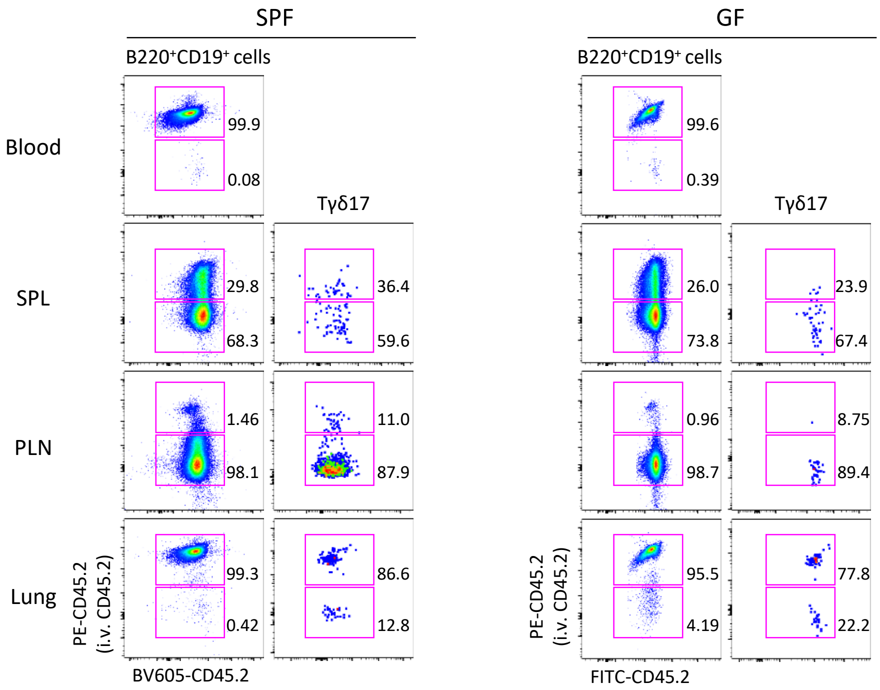

Supplementary Figure 7. Commensal microbiome does not affect circulating patterns of intravascular and extravascular cells. B6 SPF and GF mice were intravenously (i.v.) stained with anti-CD45.2-PE antibody and analyzed at 3 min after i.v. injection. Representative dot plots show B220$^+$CD19$^+$ cells and Tγδ17 cells of indicated organs. Numbers indicate frequencies of cells in adjacent gates. SPF, specific pathogen free; GF, germ-free; SPL, Spleen; PLN, peripheral lymph node.

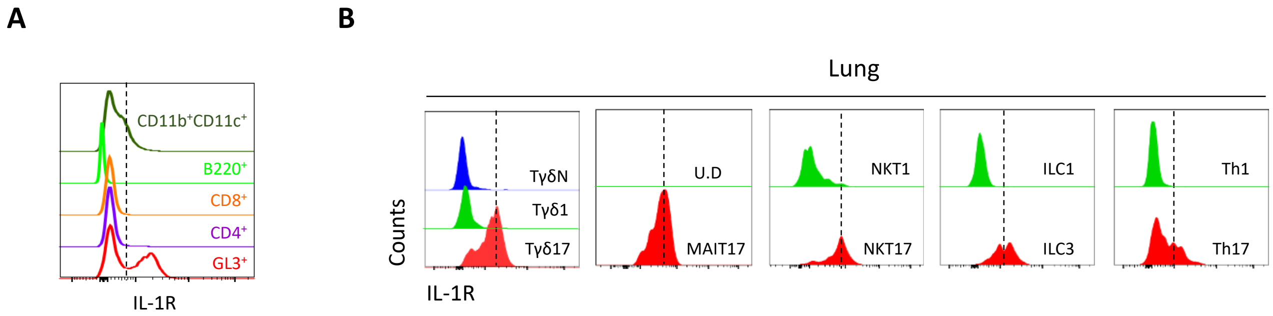

Supplementary Figure 8. Type 17 innate T cells express IL-1R in the lung. Histograms show expression levels of IL-1 receptor (IL-1R) in indicated cells at the steady state in the lung. U.D, undetected.

A

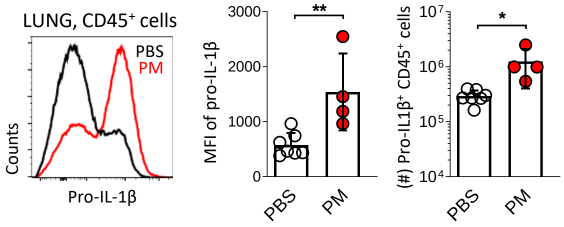

B

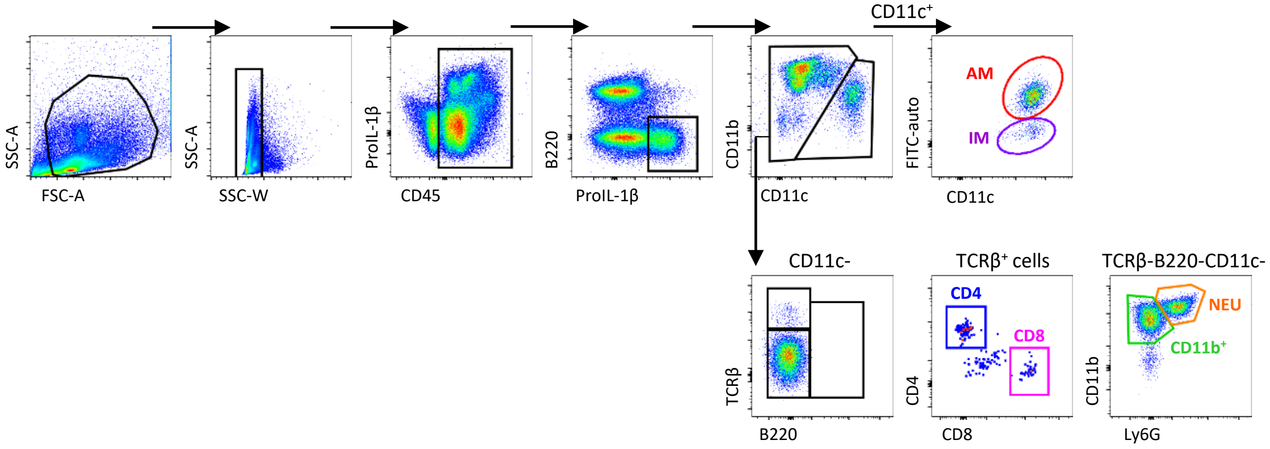

Supplementary Figure 9. PM induces IL-1β secretion from pulmonary CD45⁺ cells. (A) B6 mice were intranasally administered with 250 μg of PM or PBS and single cell suspensions of lung tissue were analyzed at 4 hours after PM exposure. Histogram shows mean fluorescence intensity (MFI) of pro-IL-1β in $CD45.2^+$ cells. Bar graphs show statistical analysis of their MFIs and absolute numbers. **(B)** Single cell suspension of lung parenchymal tissue of B6 mice were analyzed at steady state. Representative dot plots show gating strategies for indicated cell types. Each dot represents an individual mouse and horizontal bars show mean values. Data are presented as mean ± SD. Unpaired two-tailed *t*-test was used. *P <0.05, **P <0.01. AM, alveolar macrophage; IM, interstitial macrophage; NEU, neutrophil.

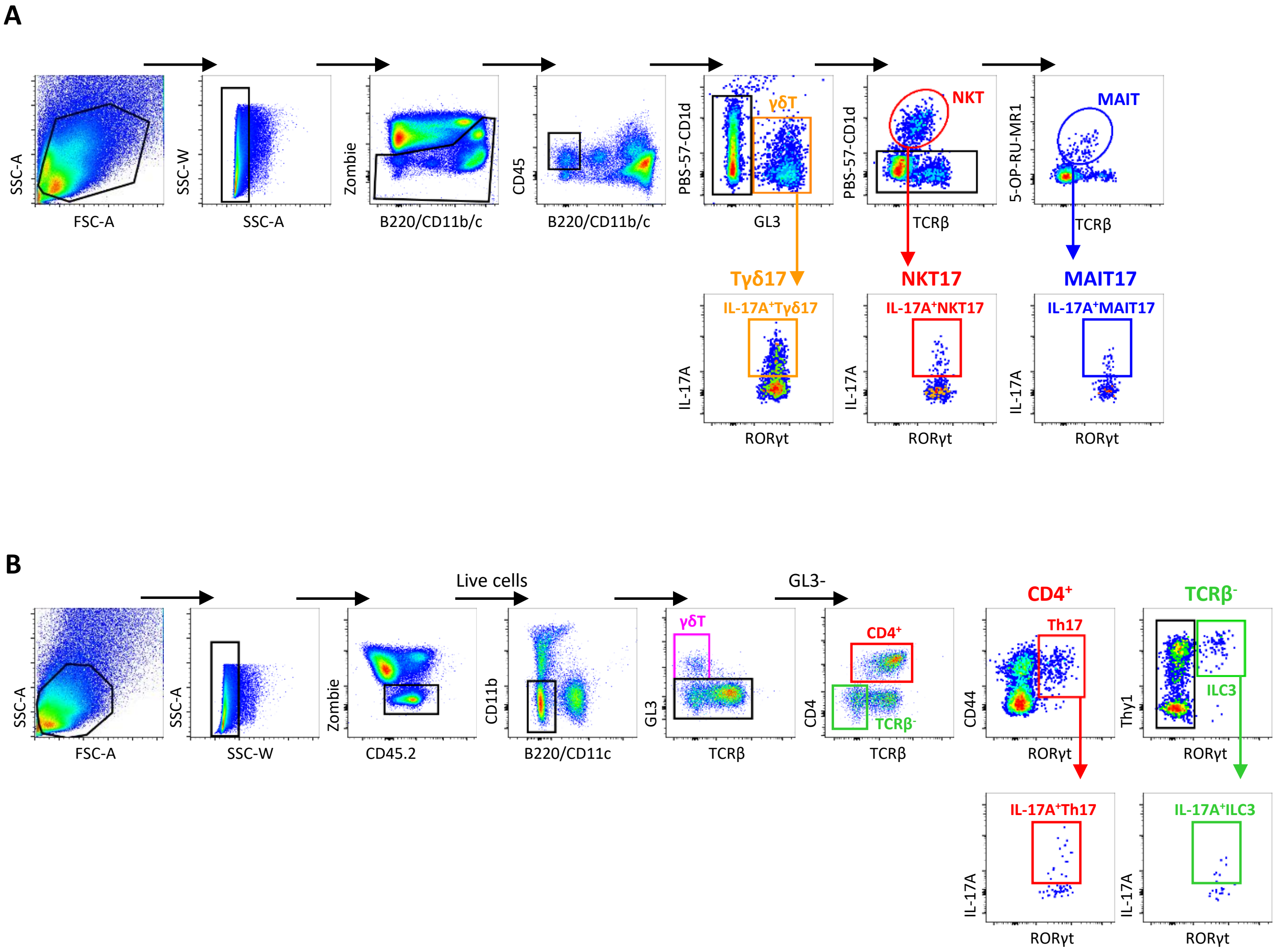

Supplementary Figure 10. Particulate matter (PM) induces acute neutrophilia and the activation of Tγδ17 cells. B6 mice were intranasally administered with 250 µg of PM and single cell suspension of lung parenchymal tissue were analyzed at 24 hours after PM exposure. **(A-B)** Representative dot plots show gating strategies for MACS enriched IL-17-producing pulmonary innate T cells (A) and ILC and conventional αβ $CD4^+$ T cells (B).

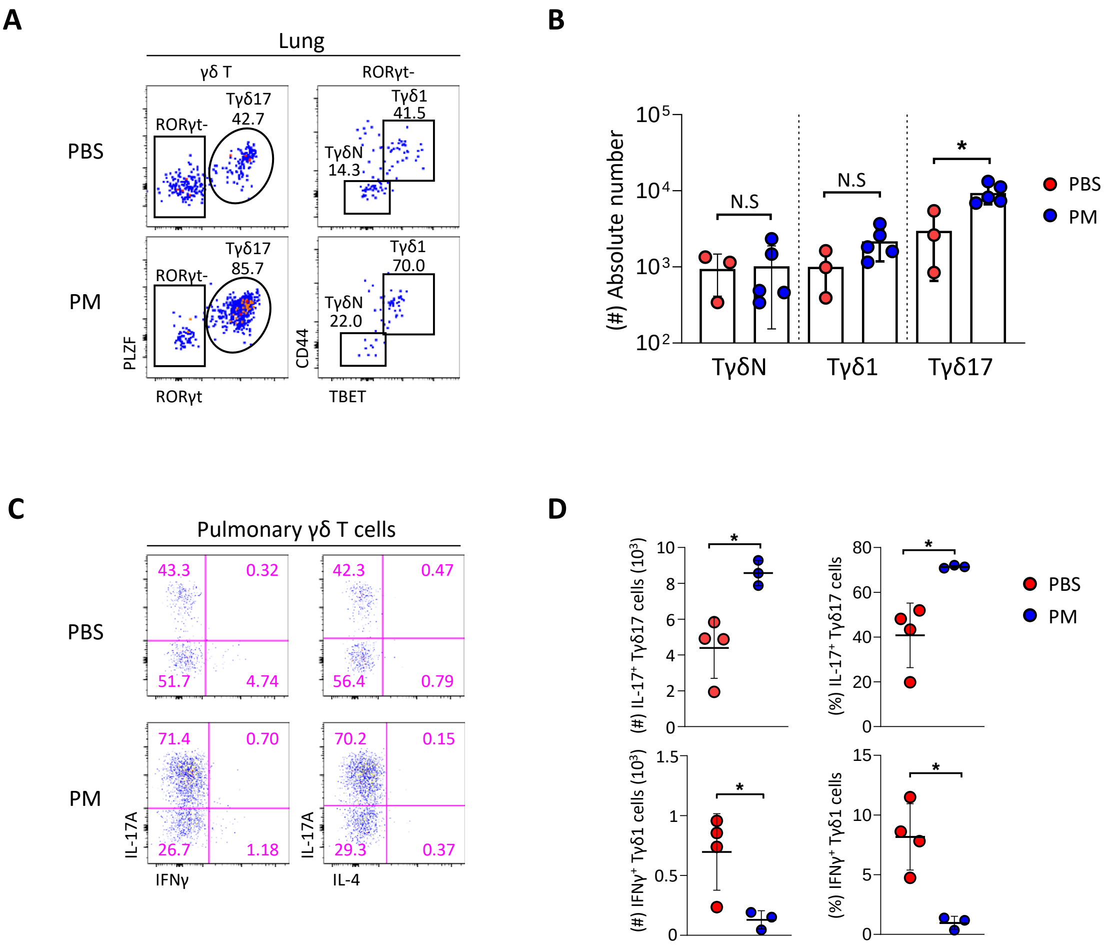

Supplementary Figure 11. PM specifically activates IL-17-producing Tγδ17 cells. B6 mice were intranasally administered with 250 μg of PM for consecutive 4 days and single cell suspension of lung parenchymal tissue were analyzed at 24 hours after last PM administration. **(A)** Representative dot plots show Tγδ17, Tγδ1 and TγδN cells. **(B)** Graphs show statistical analysis of absolute numbers of TγδN, Tγδ1, and Tγδ17 cells. **(C)** Representative dot plots show IL-17A-, IFNγ- ,and IL-4-producing γδ T cells. **(D)** Bar graphs show statistical analysis of absolute numbers and frequencies of IL-17A- and IFNγ-producing γδ T cells. Numbers indicate frequencies of cells in adjacent gates. Each dot represents an individual mouse and horizontal bars show mean values. Data are presented as mean ± SD. Unpaired two-tailed *t*-test was used. *N.S: not significant*, **P* <0.05.

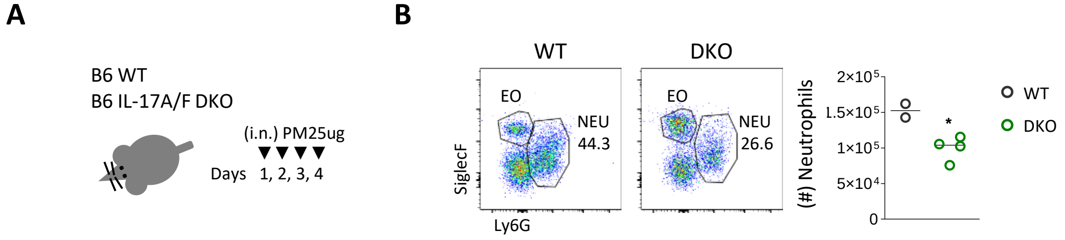

Supplementary Figure 12. IL-17 is associated with the pathogenesis of PM-induced airway neutrophilic inflammation. B6 WT and *Il17a/f* double knock-out (DKO) mice were intranasally administered with 25 μg of PM for 4 consecutive days and analyzed at day 5. **(A)** Experimental scheme is shown. **(B)** Single cell suspension of lung parenchymal tissue were analyzed using flow cytometry. Representative dot plots are shown after gating $CD11b^+$ cells. Graph shows statistical analysis of absolute number of neutrophils in lung. Numbers indicate frequencies of cells in adjacent gates. Each dot represents an individual mouse and error bars indicate ± SD*.* Unpaired two-tailed *t*-test was used. *$*P < 0.05$.* EO, eosinophil; NEU, neutrophil.
